# Delayed Intracerebral Hematoma after Ventriculoperitoneal Shunt in the Context of Ruptured Brain Arteriovenous Malformation: A Literature Review

**DOI:** 10.3390/brainsci13081159

**Published:** 2023-08-03

**Authors:** Guillaume Dannhoff, Salvatore Chibbaro, Charles-Henry Mallereau, Mario Ganau, Martial Agbo-Ponzo, Marie des Neiges Santin, Irène Ollivier, Raoul Pop, François Proust, Julien Todeschi

**Affiliations:** 1 Department of Neurosurgery, Strasbourg University Hospital, 67000 Strasbourg, France; 2 Department of Interventional Radiology, Strasbourg University Hospital, 67000 Strasbourg, France

**Keywords:** hydrocephalus, brain hemorrhage, arteriovenous malformation, ventriculoperitoneal shunt

## Abstract

Hemorrhagic complications arising from ventricular drainage procedures are typically asymptomatic and of low volume. A particular subset of these complications, known as delayed intracranial hemorrhage (DICH), is however recognized for its particularly poor prognosis. We primarily aimed to identify epidemiological characteristics associated with DICH, to shed light on its occurrence and potential risk factors. To do so, we performed a retrospective analysis of a series of ten patients who presented with DICH in the context of a ruptured brain arteriovenous malformation (bAVM) and a systematic literature review of all DICH cases reported in the literature. Our ten patients showed delayed neurological deterioration after a ventriculoperitoneal shunt (VPS) procedure, with a computed tomography (CT) scan revealing a DICH surrounding the ventricular catheter, distinct and away from the nidus of their previously ruptured bAVM. Four patients (40%) rapidly declined and passed away, three (30%) required surgical management and the remaining three (30%) demonstrated gradual clinical improvement with conservative management. In the literature, most patients presenting with DICH had hydrocephalus associated with neurovascular disorders (47% of cases), such as bAVM rupture in our present series. These constatations point out the significance of the underlying pathologies potentially being predisposed to these unusual complications.

## 1. Introduction

Treating hydrocephalus with shunt insertion is one of the most common neurosurgical procedures, performed daily on patients with various underlying pathologies. Hydrocephalus notably represents a frequent complication arising from a bleeding bAVM, with an approximate occurrence rate of 18% [1,2]. The management of this condition often necessitates the immediate placement of an external ventricular drain (EVD), followed by the potential requirement for a VPS in cases of chronic hydrocephalus [3]. Notably, instances of EVD-related hemorrhagic complications in patients undergoing hydrocephalus treatment are predominantly documented during the initial post-procedural phase [4]. Furthermore, these occurrences typically manifest as asymptomatic and small-sized (less than 5 cc) bleeds, accounting for approximately 98% of reported cases [5,6].

Recent clinical investigations have shed light on a distinct and noteworthy pattern of hemorrhagic complications termed delayed intracranial hemorrhage (DICH). DICH has been described for the first time in 1985 [7], with 52 cases reported in the literature to date. DICH encompasses instances of brain bleeding occurring in the vicinity of the ventricular catheter trajectory, surpassing a time frame of 24 h following the EVD/VPS procedure, with the initial post-operative CT-scan appearing unremarkable [8,9,10,11,12,13,14]. Intriguingly, DICH is associated with a markedly unfavorable prognosis [8,9] and currently remains misunderstood. It appears that hemorrhagic complications linked to EVD/VPS interventions show a higher incidence in patients affected by cerebrovascular diseases [5].

Within the scope of this study, we present a clinical series of 10 consecutive cases of DICH, in which we meticulously analyzed the clinical characteristics and long-term outcomes. Additionally, we conduct a systematic review of the existing literature, aiming to identify potential contributing factors that may influence the occurrence of such complications.

## 2. Materials and Methods

To delineate at best any possible underlying mechanism favoring DICH occurrence, we report an original cohort of patients presenting with DICH, along with a systematic literature review of all cases of DICH published in the literature to gather epidemiological insights.

### 2.1. Original Clinical Series

We conducted a retrospective analysis on a prospectively built database comprising 10 patients harboring a ruptured bAVM, who presented with a catheter-related DICH over a 10-year period, specifically from 1 January 2012 to 31 December 2021. Upon admission, all patients underwent an urgent brain CT scan and digital subtraction angiography (DSA), along with a systematic brain check CT scan within 24 h following the placement of the EVD/VPS, as well as in the event of clinical deterioration.

#### 2.1.1. Assessment Tools

The clinical assessment of patients on admission was performed using the Glasgow Coma Scale (GCS) [15], and their outcomes were evaluated at 3, 6, and 12 months using the Glasgow Outcome Scale (GOS). The GOS category number was assigned based on the initial scaling system described by Jennett and Bond, with a score of 1 indicating death and 5 indicating the most favorable outcome [16]. Brain hemorrhages were classified using the Fisher scale [17] and bAVM using the Spetzler/Martin scale [18]. All results were reported as median and average values, with the range and confidence interval calculated when appropriate. Qualitative variables were described using absolute and relative frequencies, while quantitative variables were presented as appropriate.

#### 2.1.2. Ethics

This study was conducted according to the Ethical Principles for Medical Research Involving Human subjects stated in 2004 and its further revisions made in 2008 and 2013 of the Declaration of Helsinki. To report our results, we followed the recommendations of the STROBE (Strengthening the Reporting of Observational Studies in Epidemiology) statement for observational studies [19]. The study was approved by the IRB committee of the French National Neurosurgery society (reference n°IRB00011687).

### 2.2. PRISMA Systematic Literature Review

**Figure 1:**
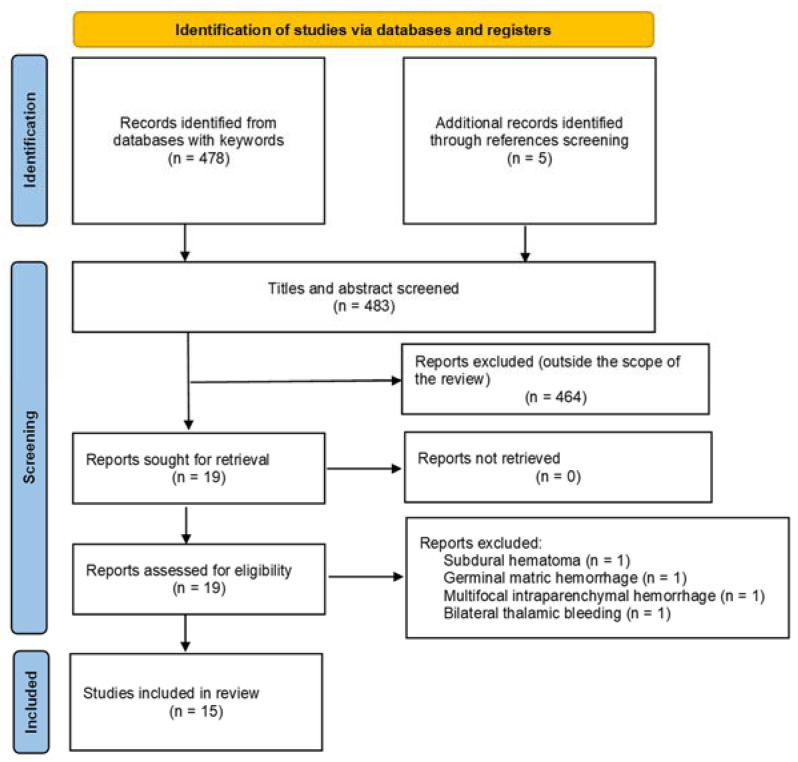
Flow chart showing selection of the studies available in the literature for the present review, according to the PRISMA statement.

To address the pertinent clinical matter, a systematic literature review was meticulously conducted in accordance with the PRISMA statement [20]. The PubMed and Ovid MEDLINE electronic databases were thoroughly searched. A comprehensive search strategy involving MeSH (Medical Subject Headings) terms and keywords was implemented, utilizing the Bolean operator “AND”. The keywords employed were “ventriculoperitoneal shunt” and “intracranial hemorrhage”, which enabled the identification of a total of 478 studies. The titles and abstracts of these studies were carefully assessed to identify those of interest. Studies lacking a clear definition and description of epidemiological data pertaining to patients affected by DICH following EVD/VPS were excluded from the final analysis. Additionally, studies detailing diverse presentations of hemorrhage were further scrutinized to identify any relevant articles on the specific topic, resulting in the inclusion of 5 additional pertinent articles. The selection of studies was performed in accordance with the PRISMA flow chart, as illustrated in Figure 1. Notably, few series reported on both early and delayed intracranial hematomas, and within the latter group, we exclusively included patients meeting the defined criteria for DICH [9].

## 3. Results

### 3.1. Clinical Series

During a 10-year period, a total of 153 patients with a ruptured bAVM were managed at our institution and 32/153 (21%) underwent VPS for the management of chronic hydrocephalus. Among the latter, 10 patients presented a catheter-related DICH; there were 4 men and 6 women, with a median age of 30 years (ranging from 21 to 61 years). These 10 patients were admitted as emergencies to our intensive care unit (ICU) due to the sudden onset of the hydrocephalus following intraventricular hemorrhage resulting from the rupture of the bAVM. It is noteworthy that all bAVM nidus locations were situated at a considerable distance from the ventricular pathway, as outlined in Table 1.

**Figure 2:**
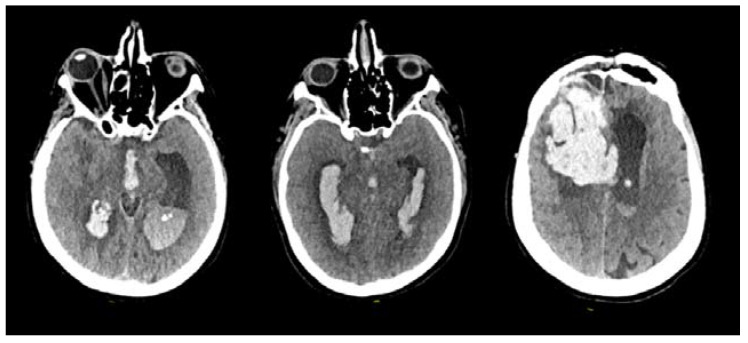
Initial CT scans of three different patients showing various intraventricular hemorrhagic extension patterns due to a ruptured bAVM.

The initial CT scan revealed the presence of an intraparenchymal hematoma combined with intraventricular hemorrhage in all patients (Figure 2). On admission, the median GCS score was 8/15 (ranging from 4/15 to 8/15), necessitating urgent intubation and sedation. Subsequently, a standard frontal EVD was expeditiously inserted. Following EVD placement, all patients exhibited varying degrees of progressive neurological improvement. However, it is important to emphasize that each patient necessitated either one or multiple EVD placements due to catheter blockage. No other complications, particularly meningitis or ventriculitis, were documented throughout the course of treatment.

**Figure 3:**
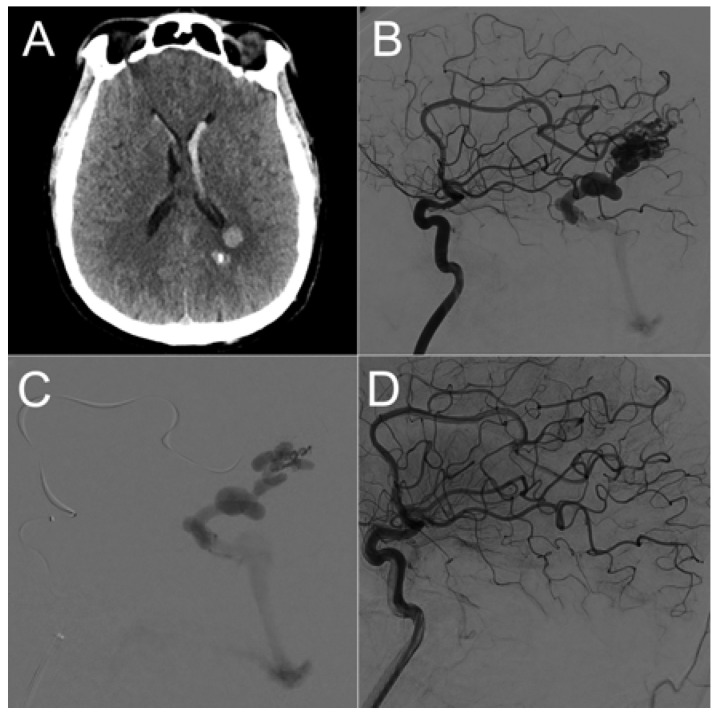
Male patient in his 40s presenting with acute onset of headache. Emergency brain CT scan (**A**) revealed left parietal intraparenchymal hematoma and intraventricular hemorrhage. Digital subtraction angiography of the left internal carotid artery (ICA) lateral projection (**B**) showed an underlying left parietal bAVM. The two main arterial feeders originated from the pericallosal artery. Endovascular embolization was performed via distal microcatheterization of the feeders and ultra-selective angiography (**C**) followed by an injection of the embolic product (Onyx 18, Ev3 Neurovascular, Irvine, CA, USA). The final angiographic of the left ICA (**D**) shows complete bAVM exclusion.

Six patients underwent endovascular management (Figure 3), two patients underwent surgical intervention, and two patients underwent a combined procedure involving embolization followed by surgery on the same day.

Treatment of the hydrocephalus was consistently approached following the management of the bAVM and once hemorrhage had been completely resolved, with cerebrospinal fluid (CSF) parameters indicating suitability for shunt insertion.

**Figure 4:**
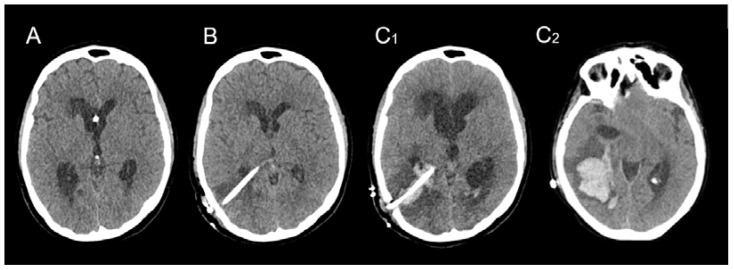
Illustrative case vignette of one patient’s consecutive CT scans: preoperative scan (**A**), early post-operative check within 24 h (**B**) and delayed (5 days) scan after neurological deterioration (**C_1_,C_2_**) showing hematoma at the level of the catheter (**C_1_**) and extension below (**C_2_**).

Following the initial hemorrhagic event, all patients underwent a VPS procedure to address chronic hydrocephalus. The median time interval between the initial hemorrhagic event and VPS placement was 25 days, ranging from 19 to 69 days. A preoperative CT scan revealed dilated ventricles without any residual signs of hemorrhage (Figure 4A). None of the patients exhibited apparent risk factors for hemorrhage, and blood clotting tests yielded normal results. Prior to surgery, all patients received low molecular-weight heparin (LMWH) prophylaxis, in accordance to our institutional protocol [21,22,23,24] for deep venous thrombosis, which was discontinued one day before the procedure. Standard VPS procedures were performed for all patients, with the ventricular catheter inserted into the right posterior ventricular horn. Adhering to our institutional policy utilizing perioperative imaging-guided system (IGS), successful and uneventful procedures were conducted, requiring only a single attempt for ventricular catheter insertion in all 10 cases. No abnormal bleeding or other perioperative issues were recorded. A Sophysa^®^ ventricular catheter measuring 6 cm in length, with an internal diameter of 1.5 mm and an external diameter of 3.0 mm, was utilized. Following the procedure, all patients recovered consciousness without any complications. Routine post-operative head CT scans were performed within 24 h after surgery, confirming the accurate positioning of the ventricular drain and the absence of hemorrhage (Figure 4B). LMWH prophylaxis was reintroduced following confirmation of a normal post-operative CT scan.

Subsequent to the initial procedures, a follow-up CT scan was performed in all patients after a few days, prompted by neurological deterioration. These scans revealed the presence of a substantial intraparenchymal hematoma surrounding the ventricular catheter, distant from the bAVM nidus (Figure 4C_1_,C_2_), along with varying degrees of intraventricular hemorrhagic extension.

Considering this progression, four patients (40%) experienced rapid deterioration leading to death in the subsequent days. In contrast, three patients (30%) underwent surgical revision of the VPS, accompanied by a temporary EVD stage also receiving intraventricular fibrinolysis. This intervention resulted in a gradual and significant neurological improvement. The remaining three patients (30%) showed progressive neurological improvement with conservative management.

### 3.2. Literature Review Analysis

Through our comprehensive systematic literature review (Table 2), we have identified several distinctive epidemiological characteristics among the 62 reported cases of catheter-related DICHs [7,8,9,10,11,12,13,14,25,26,27,28,29,30], as outlined below:

1. The median delay between the EVD/VPS procedure and the hemorrhagic complication was 5 days (range from 2 to 15 days).
2. DICH patients were rather young, with a median age of 61 years (range from 17 to 84 years).
3. A slight male predominance was noted, with a gender ratio of 1.3 M/F.
4. The most prevalent underlying pathologies in DICH patients were as follows: (a) neurovascular disorders accounted for 47% of the cases, including conditions such as spontaneous intracerebral hematoma and subarachnoid hemorrhage resulting from ruptured vascular malformations. (b) Traumatic brain injuries constituted 23% of the cases. (c) Normal-pressure hydrocephalus was observed in 20% of the cases. (d) Other pathologies, such as brain tumors and central nervous system infectious diseases, were less frequently reported, with respective incidences of 5% and 3% of the patient population.
5. The majority of DICH cases were symptomatic, accounting for 66% of patients. This symptomatic presentation correlated with an unfavorable prognosis in 44% of cases, with a Glasgow Outcome Scale (GOS) score of 3 or less, indicating poor outcomes. Surgical management was pursued for 22% of all patients, while 3% unfortunately succumbed to their condition before a surgical procedure could be performed. Conservative medical management or therapeutic abstention were approaches adopted for most patients.

## 4. Discussion

### 4.1. Hemorrhagic Complications in the Ventricular Shunting Procedure

The occurrence of asymptomatic hemorrhages following EVD or VPS procedures is a common radiological finding, particularly when performing systematic post-surgical check CT scans. In fact, after ventricular drainage, such hemorrhages can be observed in as high as one-third of all shunting procedures [6]. Hemorrhagic complications have been identified in 20.5% of EVD patients and 43.1% of VPS patients, respectively, based on systematic post-operative CT scans conducted on large patient series [31]. However, the incidence of symptomatic hemorrhagic complications leading to neurological changes is considerably lower, with an estimated 1.4% for all EVD cases and 2.9% for all VPS cases [31].

**Figure 5:**
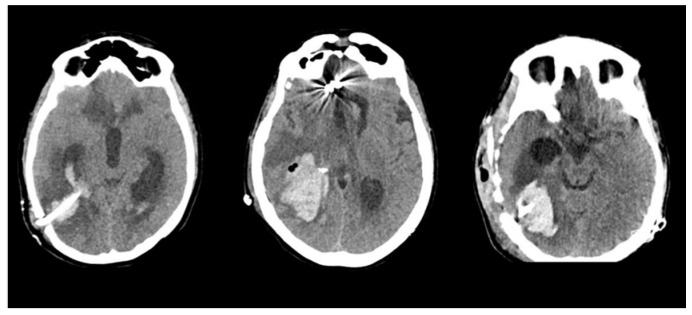
Illustrative scans of three patients of the present series, showing the unusual complication of a delayed consistent hematoma surrounding the catheter pathway, and corresponding clinically to frank neurological deterioration.

The present clinical series allowed us to draw a few considerations. All hemorrhagic complications observed in our series exhibited unusual characteristics in terms of timing, volume, and topography, as illustrated in Figure 5:

1. The location of the hematoma was consistently distant from the arteriovenous malformation (AVM) nidus, surrounding the trajectory of the ventricular catheter.
2. The occurrence of the hemorrhagic complication transpired several weeks after the initial AVM rupture and several days after the placement of the VPS.
3. Catheter-related DICH manifested in all 10 cases without any evident coagulation disorders or other identifiable risk factors.

It is of note that all VPSs were placed in the right posterior ventricular horn in our series. The proximity of the catheter with the choroid plexus could be advocated as a potential favoring factor of DICH leading to preferring a frontal shunt insertion in this subgroup of patients. However, no clear association was found between catheter localization and DICH occurrence in the literature.

On the contrary, the literature has identified several risk factors associated with early hemorrhagic complications related to the placement or removal of ventricular drains [3,5,32]. These risk factors include:

1. Age older than 75 years;
2. Anticoagulation/antiplatelet therapy;
3. Other coagulation disorders;
4. Iterative manipulations during surgery (many drain insertion attempts) surgical difficulties;
5. Larger diameter of the inserted catheter;
6. And postoperative valve manipulation/pressure changing.

Moreover, anti-platelet medication has been unequivocally identified as a significant risk factor in several series [31]. However, in our series, none of these features were observed. Thus, catheter-related DICH differs from the classically described early hemorrhagic complications, as it does not appear to be associated with anticoagulation/antiplatelet therapy, liver disease, diabetes, or hypertension, as reported by Gong et al. [8]. Most reports described patients with normal coagulation blood test results.

A study focusing on EVD procedures suggested a higher prevalence, reaching up to 39%, of hemorrhagic complications in cases of an acute hydrocephalus associated with underlying cerebrovascular diseases [6]. This group primarily consists of patients with microaneurysm rupture or hypertensive spontaneous intracerebral hemorrhage, rather than AVM rupture. However, the latter scenario suggests a propensity for locally disrupted brain coagulation systems associated with cerebrovascular diseases. The mechanisms underlying early hemorrhages following shunting procedures differ from those of DICH and are often linked to intraoperative manipulations or difficulties, whereas DICH appears to be more closely associated with an underlying primary brain disorder [29].

### 4.2. DICH

Catheter-related DICH following VPS procedures has been estimated to affect approximately 2.3% to 4% of patients [12,13]. This rate remains low in the absence of multiple drain insertion attempts, injuries to the choroid plexus, or misplacement of the catheter within the brain parenchyma [13]. Although rare, DICH is a life-threatening complication, with the mortality rate reaching as high as 58% [10].

A wide range of circumstances leading to DICH have been reported, including head injuries occurring after the procedure, bleeding from an underlying vascular malformation or cerebral lesion, coexisting bleeding disorders such as disseminated intravascular coagulation, abrupt variations in intracranial pressure after the setting and manipulation of the valve system, or even due to Valsalva’s maneuver [7,13,25,32]. However, many reports have failed to identify any clear underlying risk factors or pathological mechanisms. An interesting report even emphasized how DICH affects patients without coagulation disorders or any obvious bleeding risk, highlighting the differences in mechanisms between DICH and conventional hemorrhagic complications [12].

The most widely accepted hypothesis suggests a progressive vascular erosion and inflammatory vasculitis reaction resulting from the close contact between the catheter and the brain vessels, potentially in the context of a degenerative brain parenchymal disease [10,14,25,27]. A recent study identified an inflammatory parameter, the neutrophil-to-lymphocyte ratio, as a successful predictor of DICH following VPS, with an incidence of 40.6% in the identified subgroup, indicating the potential involvement of inflammatory pathways [33]. In a relevant series of 20 patients, Guo et al. identified statistically significant independent risk factors for DICH, including age, history of craniotomy, and radiological features on initial CT scans [9]. Additionally, the prognosis of DICH patients was statistically associated with hematoma size and initial neurological status.

It is evident that although diverse, the hypotheses proposed in the literature to explain DICH tend to converge on a substantial underlying fragility of the brain parenchyma and vessels in patients affected by primary degenerative disorders, possibly involving associated local coagulopathy or angiopathy. Considering that patients with a bAVM seem to be particularly affected by DICH, this report, by contributing new cases to the existing body of literature, allows for drawing the following pathophysiological hypotheses.

### 4.3. Arteriovenous Malformations and Physio-Pathological Mechanisms

As our understanding of cellular and molecular mechanisms underlying bAVM formation and growth improves, several hypotheses can be proposed to elucidate the physio-pathological mechanisms involved in these atypical, delayed hematomas. Pertinent research has demonstrated increased endothelial cell turnover in bAVMs [34]. The involvement of proangiogenic factors such as vascular endothelial growth factor (VEGF) and transforming growth factor (TGF) has been observed in the formation and remodeling of bAVMs [35,36]. Inflammatory reactions and angiogenic pathways are known to play a role in the hemorrhagic presentation of bAVMs [37]. Studies have shown that alternatively activated macrophages contribute to the angiogenesis of the peri-nidal-dilated capillary network and hemorrhagic foci of bAVMs [38]. These cellular and molecular findings support the hypothesis of a dynamic nature of bAVMs, with vascular and inflammatory phenomena occurring within the nidus and its surrounding regions. In light of the present observations, it is plausible that these dynamic pathways could also impact brain areas distant from the nidus and peri-nidal region, potentially causing a global parenchymal coagulopathy or angiopathy that could be further exacerbated by the inflammatory reaction triggered by the insertion of the silastic tube.

### 4.4. Hydrocephalus Management in Ruptured bAVM Patients

Our clinical series study sheds light on the potential for unusually severe hemorrhagic complications following common procedures such as VPS in patients with a history of ruptured bAVMs. Neurosurgeons should effectively communicate and inform patients and their families about these risks, as they have the potential to result in catastrophic morbidity.

Furthermore, our findings raise questions regarding the adaptation of therapeutic strategies in the management of the hydrocephalus in these patients. The consideration of alternative approaches such as lumbo-peritoneal shunts where applicable may be warranted to minimize the impact of potential hemorrhagic complications. Additionally, the assessment of perioperative medications should be meticulous, with a potential role for anti-inflammatory (steroid) medications or tranexamic acid, although further research is needed to investigate their efficacy and safety in this context.

### 4.5. Limitations

The present study has a few limitations, and notably its retrospective design, as well as its small cohort of patients. Our study voluntarily focused on patients with ruptured bAVMs as they represented, in our experience, an over-risk subgroup. Of course, the subject would benefit from further prospective multicenter studies analyzing all patients undergoing hydrocephalus management, to delineate the precise occurrence and related risk factors of DICH. Although we proposed some pathophysiological hypotheses, these should be considered cautiously as the causality remains to be demonstrated and the mechanisms at stake to be studied in detail. We hope to shed light on the subject and call for subsequent studies.

## 5. Conclusions

Patients with a bAVM are apparently prone to symptomatic hemorrhagic complications following ventricular drainage procedures, as already reported, and demonstrated in the literature, and confirmed by our current series. These findings suggest that a bAVM represents a dynamic vascular lesion with implications on a broader scale, potentially leading to a global cerebral coagulopathy and/or vasculopathy. These mechanisms may explain the increased risk of delayed cerebral bleeding occurring away from the nidus. While the exact underlying mechanisms remain unclear, caution should be exercised when considering ventricular drainage in such patients, even at a considerable time interval from the initial rupture event. Clinicians should be mindful of such potential heightened risk of hemorrhage and ensure that appropriate information is provided to the patient and their relatives prior to surgery. Furthermore, it would be of great value to investigate the potential role of anti-inflammatory drugs, such as steroids, in reducing any inflammatory component of this phenomenon through larger multicentric, randomized studies.

## Figures and Tables

**Table 1:** Epidemiological characteristics of ruptured brain arteriovenous malformations within the present series.

Sex	Age	AVM Location	Spetzler–Martin Grade	Draining Vein(s)	Treatment
M	21	Left parieto-occipital	3	Lateral sinus	Embolization
M	40	Left parietal	3	Straight sinus	Embolization
F	37	Left cerebellar	3	Sigmoid sinus	Embolization
F	58	Right cerebellar	3	Vein of Galen, inferior petrous sinus	Embolization
F	29	Right parietal	3	Superior sagittal sinus	Surgery
M	30	Right parietal	2	Superior sagittal sinus	Surgery
F	57	Right frontal	3	Vein of Galen	Embolization
M	61	Left basifrontal	3	Vein of Galen	Embolization
F	48	Left temporo-parietal	2	Superior sagittal sinus	Embolization + Surgery
F	34	Left temporal	3	Superior sagittal sinus, lateral sinus	Embolization + Surgery

**Table 2:** Epidemiological characteristics of DICH in cases reported in the literature.

Series	Number of Patients	Onset Day of Hemorrhage	Age	Gender	Localization of the Ventricular Catheter	Primary Disease Leading to Hydrocephalus	Symptomatic	Management	Glasgow Outcome Scale
Matsumura et al. (1985) [7]	1	7	17	M	AH	TBI	Yes	Surgery	5
Snow et al. (1986) [25]	1	7	43	F	AH	NPH	Yes	-	-
Derdeyn et al. (1988) [26]	2	2	56	M	PH	TBI	Yes	Surgery	4
		2	73	F	AH	NPH	Yes	Conservative	4
Mascalchi et al. (1991) [27]	1	15	68	M	AH	ICH	Yes	Conservative	1
Savitz et al. (1999) [13]	2	2	-	-	PH	-	No	Conservative	-
		2	-	-	PH	-	No	Conservative	-
Alcazar et al. (2007) [28]	1	6	64	F	PH	SAH	Yes	Surgery	1
Misaki et al. (2010) [29]	2	5	55	M	PH	SAH	No	Conservative	-
		3	64	M	PH	SAH	No	Conservative	-
Zhou et al. (2012) [11]	2	5	32	F	AH	NPH	Yes	Death before surgery	1
		3	58	M	AH	TBI	Yes	Conservative	4
Ma et al. (2015) [10]	1	8	67	M	AH	TBI	Yes	Palliative care	-
Guo et al. (2017) [9]	20	3	58	F	-	SAH	Yes	Conservative	4
		3	54	M	-	ICH	Yes	Conservative	1
		3	61	M	-	TBI	Yes	Conservative	3
		4	61	M	-	Tumoral	Yes	Conservative	1
		4	75	M	-	ICH	Yes	Conservative	5
		5	84	F	-	TBI	Yes	Conservative	5
		6	48	F	-	SAH	Yes	Surgery	1
		6	61	M	-	NPH	Yes	Surgery	1
		6	62	M	-	TBI	Yes	Surgery	2
		6	78	M	-	NPH	No	Conservative	5
		7	64	F	-	SAH	Yes	Conservative	4
		7	65	F	-	Tumoral	No	Conservative	5
		7	76	F	-	ICH	Yes	Surgery	2
		8	66	M	-	TBI	No	Conservative	5
		8	69	M	-	NPH	No	Conservative	5
		9	57	F	-	SAH	Yes	Conservative	5
		9	69	M	-	NPH	Yes	Conservative	5
		9	72	M	-	NPH	Yes	Conservative	5
		10	33	M	-	ICH	Yes	Conservative	5
		10	30	M	-	ICH	Yes	Conservative	4
Gong et al. (2017) [8]	12	3	62	M	AH	ICH	Yes	Death before surgery	1
		3	64	F	PH	TBI	-	Conservative	3
		7	76	M	AH	SAH	-	Conservative	3
		3	50	M	AH	SAH	Yes	Surgery	2
		4	61	F	AH	TBI	-	Conservative	4
		5	67	M	AH	Infectious	-	Conservative	3
		7	65	M	AH	NPH	No	Conservative	4
		4	61	M	AH	NPH	-	Conservative	5
		3	60	M	AH	TBI	-	Conservative	4
		4	53	F	PH	SAH	-	Surgery	2
		5	68	F	AH	NPH	-	Conservative	5
		5	61	M	AH	SAH	-	Surgery	3
Hou et al. (2017) [12]	4	9	56	F	AH	Tumoral	Yes	Surgery	1
		2	48	M	AH	TBI	Yes	Conservative	5
		3	65	M	PH	NPH	Yes	Conservative	5
		4	51	F	AH	TBI	Yes	Conservative	1
Musali et al. (2019) [30]	1	7	56	F	PH	Infectious	Yes	Conservative	1
Wang et al. [14] (2021)	2	9	49	F	PH	SAH	Yes	Conservative	5
		6	76	F	PH	TBI	Yes	Conservative	5
Present study	10	2	21	M	PH	AVM	Yes	Conservative	4
		2	44	M	PH	AVM	Yes	Conservative	1
		3	37	F	PH	AVM	No	Surgery	4
		4	58	F	PH	AVM	Yes	Surgery	3
		5	29	F	PH	AVM	Yes	Conservative	3
		5	30	M	PH	AVM	Yes	Conservative	1
		5	57	F	PH	AVM	No	Surgery	3
		6	61	M	PH	AVM	No	Conservative	1
		6	48	F	PH	AVM	Yes	Conservative	1
		6	34	F	PH	AVM	Yes	Conservative	4

ICH: intracerebral hematoma (hypertensive), SAH: subarachnoid hemorrhage, TBI: traumatic brain injury, AH: anterior horn, PH: posterior horn, NPH: normal-pressure hydrocephalus.

## Data Availability

Not applicable.
